# Behavior of Chemokine Receptor 6 (CXCR6) in Complex with CXCL16 Soluble form Chemokine by Molecular Dynamic Simulations: General Protein‒Ligand Interaction Model and 3D-QSAR Studies of Synthetic Antagonists

**DOI:** 10.3390/life11040346

**Published:** 2021-04-15

**Authors:** Giovanny Aguilera-Durán, Antonio Romo-Mancillas

**Affiliations:** 1 Posgrado en Ciencias Químico Biológicas, Facultad de Química, Universidad Autónoma de Querétaro, Cerro de las Campanas S/N, Querétaro 76010, Mexico; giovanny.aguilera@uaq.mx; 2 Laboratorio de Diseño Asistido por Computadora y Síntesis de Fármacos, Facultad de Química, Universidad Autónoma de Querétaro, Centro Universitario, Querétaro 76010, Mexico

**Keywords:** CXCR6, CXCL16s, CG-MD simulations, docking, 3D-QSAR

## Abstract

The CXCR6‒CXCL16 axis is involved in several pathological processes, and its overexpression has been detected in different types of cancer, such as prostate, breast, ovary, and lung cancer, along with schwannomas, in which it promotes invasion and metastasis. Moreover, this axis is involved in atherosclerosis, type 1 diabetes, primary immune thrombocytopenia, vitiligo, and other autoimmune diseases, in which it is responsible for the infiltration of different immune system cells. The 3D structure of CXCR6 and CXCL16 has not been experimentally resolved; therefore, homology modeling and molecular dynamics simulations could be useful for the study of this signaling axis. In this work, a homology model of CXCR6 and a soluble form of CXCL16 (CXCR6‒CXCL16s) are reported to study the interactions between CXCR6 and CXCL16s through coarse-grained molecular dynamics (CG-MD) simulations. CG-MD simulations showed the two activation steps of CXCR6 through a decrease in the distance between the chemokine and the transmembrane region (TM) of CXCR6 and transmembrane rotational changes and polar interactions between transmembrane segments. The polar interactions between TM3, TM5, and TM6 are fundamental to functional conformation and the meta-active state of CXCR6. The interactions between D77-R280 and T243-TM7 could be related to the functional conformation of CXCR6; alternatively, the interaction between Q195-Q244 and N248 could be related to an inactive state due to the loss of this interaction, and an arginine cage broken in the presence of CXCL16s allows the meta-active state of CXCR6. A general protein‒ligand interaction supports the relevance of TM3‒TM5‒TM6 interactions, presenting three relevant pharmacophoric features: HAc (H-bond acceptor), HDn (H-bond donator), and Hph (hydrophobic), distributed around the space between extracellular loops (ECLs) and TMs. The HDn feature is close to TM3 and TM6; likewise, the HAc and Hph features are close to ECL1 and ECL2 and could block the rotation and interactions between TM3‒TM6 and the interactions of CXCL16s with the ECLs. Tridimensional quantitative structure-activity relationships (3D-QSAR) models show that the positive steric (VdW) and electrostatic fields coincide with the steric and positive electrostatic region of the *exo*-azabicyclo[3.3.1]nonane scaffold in the best pIC50 ligands. This substructure is close to the E274 residue and therefore relevant to the activity of CXCR6. These data could help with the design of new molecules that inhibit chemokine binding or antagonize the receptor based on the activation mechanism of CXCR6 and provoke a decrease in chemotaxis caused by the CXCR6‒CXCL16 axis.

## 1. Introduction

The migration of immune system cells, or chemotaxis, to maturation tissues and sites of injury is controlled by small proteins (8–14 KDa) called chemokines [1,2]. About 50 chemokines and 20 receptors have been reported, indicating that several chemokines interact with one or more receptors. These chemokines exert their effects by means of interaction with transmembrane G-protein coupled receptors (GPCRs), called chemokine receptors. The chemokines are classified into four families according to the arrangement of the first cysteines, CC, CXC, CX_3_C, and XC [3,4]. The chemokine CXCL16 (SR-PSOX) is a chemokine of 254 amino acids, which, along with CX_3_CL1 (fractalkine), has two forms: a soluble form (CXCL16s) and a membrane-anchored form (CXCL16m) [5,6]. CXCL16 is expressed in immune system cells, keratinocytes, and endothelial cells [7,8]. The isoform CXCL16m acts as a receptor for oxidized LDL, phosphatidylcholine, dextran sulfate, and bacteria; helps in the phagocytosis of bacteria; and promotes the adhesion of cells of the immune system. It contains a cytoplasmic region, a transmembrane region, a mucin-like region, and a chemokine-like region. CXCL16m is cleaved by the action of metalloproteases ADAM10 and ADAM17 to release the soluble form [9,10,11]. Chemotaxis is the main effect of the soluble counterpart (CXCL16s) [5,12].

The CXCR6 receptor, also known as Bonzo or TYMSTR, is a transmembrane GPCR [13,14] that contains a DRF sequence, in contrast to the other chemokine receptors, which have a conserved DRY sequence. This motif has been associated with the promotion of cell adhesion [8]. CXCL16 is the only known natural ligand of chemokine receptor CXCR6 [3,8,15]. This receptor is expressed in immune system cells such as natural killers, CD4^+^ and CD8^+^ lymphocytes, ILC (innate lymphoid cells), fibroblasts, smooth muscle cells, and endothelial cells [2,8,16].

**Figure 1:**
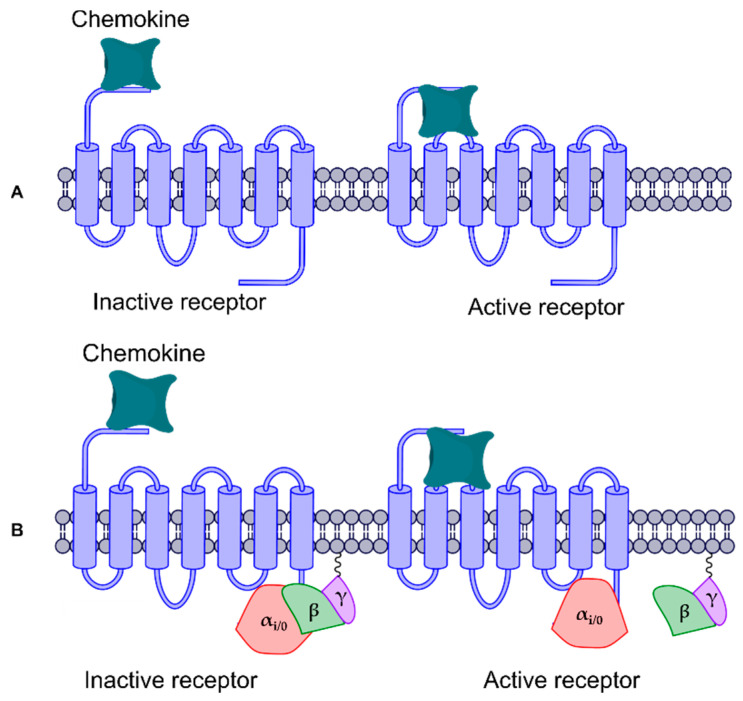
Activation mechanism of chemokine receptors. (**A**) Initial interaction of a chemokine with NH_2_-T and the movement into the transmembrane region, promoting a conformational change in the receptor; (**B**) interaction of the chemokine with NH_2_-T and movement into the transmembrane region, promoting a conformational change in the receptor and triggering the separation of the βγ complex from the α_i/0_ subunit in the G-protein.

The proposed activation of the chemokine receptors has two steps: first, the interaction of a chemokine with the amino terminal (NH_2_-T) promotes its movement into the transmembrane region; and then a change in the H-bonds between transmembrane segments (TMs) activates the receptor. Other authors have proposed the separation of the βγ complex from the α subunit in G-protein as the second activation step (Figure 1) [17,18,19,20]. Furthermore, the rotation and polar interactions of TMs are related to GPCR activation [21,22]. The DRY motif is present in most chemokine receptors; alternatively, CXCR6 has an analogous DRF motif relevant to binding different types of G_i/0_-protein, and mutation in this motif can reduce G-protein coupling and CXCR6 expression [22,23,24,25,26,27].

The CXCR6‒CXCL16 axis is involved in several pathological processes, and they are overexpressed in different types of cancer, such as prostate, breast, ovary, and lung cancer, as well as schwannomas, promoting invasion and metastasis. Additionally, this axis is involved in atherosclerosis, type 1 diabetes, primary immune thrombocytopenia, vitiligo, and other autoimmune diseases, in which it is responsible for the infiltration of different immune system cells [3,28,29,30,31,32,33,34,35]. Thus, antagonism of CXCR6 has been proposed in recent years as a relevant target for the treatment of various pathologies. However, the lack of a crystallographic structure of this receptor hinders the understanding of the molecular processes of CXCR6 agonism and antagonism; moreover, there are a few specific antagonists to CXCR6, and they have mainly been found via high-throughput screening and optimization methods [36].

In silico methodologies such as homology modeling, molecular docking, and molecular dynamics simulations are helpful when the tridimensional structure of a protein has not been resolved experimentally. Among these computational methodologies, protein homology modeling allows for an approximation of a protein to a three-dimensional structure based on the principle that “similar primary sequences will have a similar three-dimensional conformation” [20,37]. Additionally, molecular docking is used to model the interaction between two chemical entities, which can be small ligands, proteins, or nucleic acids, and molecular dynamics simulations, in order to study the vibration, torsion, and behavior of bonds, as well as non-bonding interactions such as electrostatic interactions and van der Waals forces, and to observe the evolution of a system in the presence of a ligand (small molecule, peptide, or protein) under constant conditions of pressure, temperature, volume, and energy [38,39,40].

When CXCR6 is activated by CXCL16s, nucleotide exchange is promoted (GDP for GTP) in the α_i/0_ subunit in the G-protein and dissociation of the βγ subunit from α_i/0_ subunit, and the activation of phospholipase C increases the concentrations of inositol triphosphate (IP_3_) and Ca^2+^, which increase the phosphorylation of AKT, promoting chemotaxis, proliferation, and survival [8,41]. The interaction with CXCL16m promotes conformational changes in the receptor that result in cell adhesion [15,22].

CXCR6 models have been reported using CXCR4 or CCR5 receptors as templates [8,22]. Relevant amino acids for the interaction of CXCL16s with CXCR6 have been suggested, such as D176 and E274, with mutations in these amino acids inhibiting the binding of CXCL16s. Furthermore, mutation E274Q promotes cell adhesion, showing that CXCR6 can discriminate between two forms of CXCL16 [15]. However, much remains to be studied in relation to the interactions between the different isoforms of CXCL16 and the CXCR6 receptor. Recently, the CCR6–CCL20 complex by Cryo-EM was reported (PDB code: 6WWZ). This complex constitutes a powerful tool for the future modeling of chemokine-receptor complexes, as the binding mode of the agonist CCL20 to CCR6 resembles those observed for the CXCR4–vMIP-II and US28–CX3CL complexes [42].

Thus, homology modeling of the chemokine receptor CXCR6 and the soluble form of chemokine CXCL16, as well as molecular dynamic simulations to study the interactions between CXCR6 and CXCL16, are reported in this work, along with general protein‒ligand interactions and tridimensional quantitative structure-relationship (3D-QSAR) models based on the reported antagonists. Our objective was to observe the conformational changes of CXCR6 in the presence of CXCL16s and the protein‒protein interactions present in these complexes, to better understand the activation of this receptor, and to identify pharmacophoric features in small ligands that promote binding to this receptor. The results could contribute to the design of new highly specific CXCR6 antagonists for the pathologies in which the CXCR6‒CXCL16 axis is involved, like the metastasis of cancer cells and autoimmune pathologies.

## 2. Materials and Methods

Based on previous works by our research group [20,43], the methodology was modified accordingly, as stated in the following subsections.

### 2.1. Protein Structures, Homology Modeling of CXCR6 and CXCL16s, and Relaxation by All Atom Molecular Dynamics (MD) Simulations

The primary sequence of the CXCR6 receptor and the chemokine CXCL16 were obtained from the UniProt database (codes O00574 and Q9H2A7, respectively) [44]. The amino acid sequence of CXCR6 and CXCL16s (chemokine region) were uploaded to the GPCR-I-TASSER [45] server and the general I-TASSER server, respectively [46].

The homology modeling of CXCR6 was calculated using CCR5 (PDB code: 4MBS) [47] as a template in the GPCR-I-TASSER module. For the CXCL16s, the homology modeling was calculated using CCL20 (PDB code: 2JYO) [48] as a template for the chemokine region (32‒107 a.a.) in the general module of the I-TASSER server.

In the homology model of CXCR6, the NH_2_-T was manually reoriented and further minimized because the model conformation obtained from GPCR-I-TASSER was oriented to the transmembrane region. The preparation of a system for relaxation by means of all-atom MD simulation, including the addition of post-translational modifications (disulfide bridges and glycosylations) present in proteins, was undertaken on the CHARMM-GUI web server [49,50]. MD simulation was performed with Gromacs 2018.7 [51,52], using an isothermic‒isobaric ensemble (NPT) at 1 atm and 310.15 K, employing temperature coupling and velocity rescaling with a stochastic term [53], and a Parrinello‒Rahman barostat [54]. The systems were submitted to 5000 steps of steepest-descent minimization, followed by a six-equilibration-step scheme for reducing force constants, as suggested by the CHARMM-GUI server. The TIP3 water model and POPC lipids were used. The system size was 13.354 × 13.430 × 13.357 nm, with a volume size of 2371.17 nm^3^, and with 53,579 TIP3 waters, 419 POPC lipids (upper/lower leaflet ratio 220/217), 146 Na^+^ ions, and 149 Cl^−^ ions.

For CXCL16s, the system was prepared in the solution builder module of CHARMM-GUI with added disulfide bridges, and at 5 ns all atom MD simulations were performed in Gromacs 2018.7 [51,52], using an isothermic‒isobaric ensemble (NPT) at 1 atm and 310.15 K, employing temperature coupling and velocity rescaling with a stochastic term [53] and a Parrinello‒Rahman barostat [54]. The system was submitted to 5000 steps of steepest-descent minimization, followed by the one-equilibration-step scheme for reducing force constants, as suggested by the CHARMM-GUI server, prior to performing production simulation using the TIP3 water model. The system size was 5.578 × 5.576 × 5.614 nm, with a volume size of 166.38 nm^3^, with 4726 TIP3 waters, 13 Na^+^ ions, and 18 Cl^−^ ions.

The module “cluster” of Gromacs 2018.7 was used to find the relevant conformations (cluster1) of the simulation using the “gromos” algorithm [55].

### 2.2. Protein‒Protein Docking

Molecular docking was performed using the ClusPro server [56,57,58,59] to build the CXCR6‒CXCL16s complexes, using the structure of minimization for CXCR6 and cluster1 for CXCL16s. The proximity to the first 25 residues in CXCR6 was considered a restriction for molecular docking. In the case of chemokines, no restrictions were set [60].

The ClusPro web server protocol is based on PIPER [57]. Briefly, the center of mass of the receptor is fixed at the origin coordinate system, the rotational and translational positions of the ligand are evaluated at the given level of discretization, and the rotational space is sampled on a sphere-based grid [61]. The best model is selected by clustering the population by the lowest scoring energy of 1000 docked structures using pairwise-RMSD as the distance measure for each pair among the 1000 structures and to find the structure that has the highest number of neighbors within a 9-Å radius. The selected structure is defined as the center of the first cluster, and the structures within the RMSD neighborhood constitute the first cluster. The members of this cluster are then removed and the structure with the highest number of neighbors within the radius among the remaining structures is selected as the next cluster center, and so forth. Up to 30 clusters are generated, and structures are minimized for 300 steps with a fixed backbone using the van der Waals term of the Charmm forcefield [62], removing steric overlaps but generally with small conformational changes. ClusPro outputs the structures at the center of the 10 most populated clusters, PIPER energies of cluster centers, and the lowest PIPER energy within each cluster; however, these values do not include entropic contributions and the weights of the energy components are selected to yield near-native structures, rather than correct thermodynamics of binding [57].

Seventeen models of interactions were created by ClusPro on the combination of hydrophobic and electrostatic interactions; the conformation of complex by ClusPro that was chosen for the CG-MD simulations was the one that presented the highest number of conformations in the cluster. Interactions between CXCR6 and CXCL16s were observed by means of LigPlot+ [63,64].

### 2.3. Coarse-Grained Molecular Dynamics (CG-MD) Simulations

To evaluate whether the CXCR6 model reached an activated conformation, a system for the molecular dynamics simulation of CXCR6‒CXCL16s was built using the Martini Maker module [49,65] in CHARMM-GUI. The Martini2.2p force field was used in an NPT assembly at 1 atm and 310.15 K, with a PW model of polarizable water, and POPC beads were used. Coarse-grained simulations of 1 μs were produced in Gromacs 2018.7 [51,52]. CG-MD simulations were performed on two different systems to compare a CXCR6 structure without the stimuli of chemokines and the complex between CXCR6 and CXCL16s (CXCR6 CG-MD and CXCR6‒CXCL16s CG-MD, respectively). The system size of CXCR6 CG-MD was 13.897 × 13.405 × 15.309 nm, with a volume of 2433.39 nm^3^, 437 POPC lipids (upper/lower leaflet ratio 220/217), 13,669 PW waters, 155 Na beads, and 158 Cl beads. The system size of CXCR6‒CXCL16s CG-MD was 13.259 × 13.139 × 15.818 nm, with a volume size of 2524.78 nm^3^, 437 POPC lipids (upper/lower leaflet ratio 220/217), 14,264 PW waters, 161 Na beads, and 169 Cl beads. To observe the evolution of the system, the conformations were analyzed every 100 ns with LigPlot+. All those frames were previously converted to an all-atom structure by means of the Martini Maker all-atom converter module in the CHARMM-GUI server.

Five runs of CG-MD simulations were performed for each CG system (CXCR6 CG-MD and CXCR6‒CXCL16s CG-MD) to obtain a large conformational sampling. Subsequent analyses were performed in representative structures of each 100 ns (F0–F10), which were obtained using the Average Structure of Structure Analysis module of the academic version of the Schrödinger Maestro program, which used a uniform weighting method and superimposed all structures to one entry, considering heavy atoms for RMSD and checking the symmetry in RMSD comparisons. Then, this module selected as the representative structure the one with the smallest RMSD from the average structure. The uniform weighting used the same weight for each structure and checked for local symmetry in the connectivity (such as a ring orientation), which could reduce the RMSD, and used the lowest RMSD value [66].

Calculations of the RMSD of CXCR6 free, CXCR6 in complex, CXCL16s, ICLs, ECLs, NH_2_-T, COOH-T, and TMs were performed for each CG simulation, as well as the RMSF to TMs and ICL2, and the distance between CXCL16s and CXCR6. All graphs were built with R [67] using ggplot2 libraries [68], using the average RSMD, RMSF, and distance of five CG simulations to CXCR6 CG-MD and CXCR6‒CXCL16s CG-MD. The “distance” module of Gromacs 2018.7 was used to measure the distance between the chemokine and CXCR6 [55].

### 2.4. Ligand Docking, General Protein–Ligand Interaction Model, and Receptor-Based 3D-QSAR Model

The 3D structures of 82 structures of reported antagonists of CXCR6 with IC50 values ranging from 0.04 to 50 μM [36] were modeled in the Schrödinger Maestro program. A first screening to find the binding site of antagonists was performed in F0 of CXCR6 CG-MD, and a grid of 110 × 100 × 255 Å with a spacing of 0.375 Å was calculated using AutoGrid 4.2.6 [69]. Then, molecular docking was performed with AutoDock 4.2.6(cripps Institute, La Jolla, CA, USA) optimized for GPU, using a total of 50 runs and 25,000,000 evaluations with a Lamarckian genetic algorithm and a Solis–Wets local search [70]. Using the binding site of the molecule with the best IC50, a new grid was calculated, of 80 × 80 × 80 Å with a spacing of 0.375 Å, and a second molecular docking was performed using AutoDock 4.2.6 optimized for GPU, using a total of 50 runs and 25,000,000 evaluations with a Lamarckian genetic algorithm and a Solis–Wets local search [20].

Pharmer (2015, Pittsburgh, PA, USA) [71] was used to identify the pharmacophoric elements such as hydrogen bond acceptors (HAc), hydrogen bond donors (HDn), aromatic rings (Arm), hydrophobic groups (Hph), positive-charged groups (PIn), and negative-charged groups (NIn) of the docked ligands in their most frequent binding conformation, which were obtained by clustering the results from the second docking of each ligand with a root mean standard deviation (RMSD) cutoff of 2 Å on their protein–ligand complexes [20,43]. These characteristics were clustered according to their nature, position, and size using the partitioning around medoids statistical method [72], implemented in the cluster package [73] available in the statistical software R (3.5.2, R Foundation for Statistical Computing, Vienna, Austria) [67] to build a general protein‒ligand interaction model [20].

The poses from the second molecular docking were also used as alignment suppositions to calculate the quantitative structure-activity relationship (QSAR) models using OPEN3DQSAR program [74]. The steric and electrostatic fields were calculated like Comparative Molecular Field Analysis (CoMFA) molecular fields [75], using pIC50 as a dependent variable. The leave-one-out (LOO) method was used to test the QSAR model [76], with 28% of the compounds serving as a random subset to test the models.

### 2.5. Manipulations of Complexes and Figures

The manipulation of protein complexes and antagonist modeling was performed using the academic version of the Schrödinger Maestro program [66]. All of the protein Figures presented in this article, the alignments, the analysis of TMs’ interactions, and the analysis of TMs’ rotation were made in the PyMOL program [77]. The rotation was performed using a free Python script of rotation axis “draw_rotation_axis.py” in the official repository of PyMOL scripts (https://pymolwiki.org/index.php/Pymol-script-repo, accessed on 20 January 2021). The script calculated the rotation axis value using F0‒F10 of representative structures.

## 3. Results and Discussion

### 3.1. Protein Structures and Homology Modeling

All Ramachandran plots are shown in Figure S1. The I-TASSER server built five models of CXCR6 using chain A of CCR5 with a sequence identity of 34.74%. A percentage greater than 30% facilitates the prediction of structures at the time of modeling (Figure S2A) [78]. A CXCR6 model based on the CCR5 structure is a more robust model because I-TASSER builds 3D structural models by reassembling fragments excised from threading templates starting from the amino acid sequence; then, biological insights into the target proteins are deduced by matching the structural models to known proteins in the functional databases. I-TASSER always creates 3D models for the complete sequence without discarding a single amino acid, due to ab-initio folding based on the replica-exchange Monte Carlo simulation procedures that I-TASSER applies for modeling structural segments such as loops [79].

**Figure 2:**
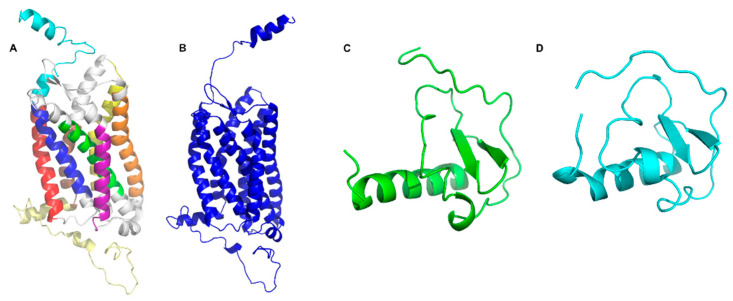
Structures of CXCR6 and CXCL16s: (**A**) CXCR6-M1. NH_2_-T is depicted in turquoise, TM1 in red, TM2 in blue, TM3 in green, TM4 in purple, TM5 in orange, TM6 in yellow, TM7 in brown, and COOH-T in pale yellow; (**B**) CXCR6 reorientated; (**C**) CXCL16s; and (**D**) CXCL16s cluster1.

I-TASSER selected the best model through analysis of the C-score (with a range of −5 to 2, where higher values represent greater confidence in the model) and TM score (greater than 0.5 is considered a model with correct topology). The best model, model 1 (CXCR6-M1, Figure 2A) had a C-score of −0.46 and a TM score of 0.65 ± 0.13. In addition, CXCR6-M1 was evaluated using the MolProbity public server [80] to obtain Ramachandran plots to validate this model. 

In the CXCR6 homology model, the percentage of residues in favorable areas was 85.29%. The higher the percentage of residues in the favored regions in the Ramachandran plot, the better the stereochemical quality of the models [81]. According to this metric, the CXCR6 model developed by I-TASSER was a suitable model, even without a relaxation process by MD; however, the NH_2_-T region in CXCR6 was oriented to the transmembrane region. This orientation did not permit us to observe a decrease in distance between the chemokine and the receptor. For this reason, the NH_2_-T was manually reoriented to “open” the receptor (Figure 2B); this new conformation was evaluated in MolProbity, with 84.71% of favored residues.

In the case of CXC16s, the I-TASSER server built five homology models using CXCL20 as a template with a sequence identity of 31.25% (Figure S2B), calculated by the BLAST server [82,83]. Model 1 (Figure 2C) had a C-score of −1.51 and a TM-score of 0.53 ± 0.15. The percentage of residues in favorable areas in the Ramachandran plot was 67.57%, suggesting that the model would need a MD simulation to improve its quality.

### 3.2. Relaxation of CXCR6 and CXCL16s

CXCR6 was only minimized using the minimization and equilibrium standard protocol suggested by CHARMM-GUI, and the conformation after minimization was aligned to CXCR6-M1, with a root mean standard deviation (RMSD) of the α-carbon of 1.202 Å (Figure S3A). The CXCR6 minimized structure was evaluated in MolProbity; the residues in favorable areas were 91.76%, indicating that the structure is suitable for subsequent simulations.

To improve the quality of CXCL16s-M1, an all-atom MD of 5 ns was performed to equilibrate the CXCL16s homology model. The most representative conformation (cluster1) of CXCL16s (Figure 2D) was aligned with the homology model, with a RMSD of 2.602 Å (Figure S3B). After the AA-MD, the percentage of favored residues of the Ramachandran plot of CXCL16s was 89.19%, showing that cluster1 of the simulation has an appropriate conformation for subsequent studies. The RSMD was used as a measure of change in the system with respect to the starting structure. Table 1 presents a summary of the quality of the CXCR6 and CXCL16s structures.

### 3.3. CXCR6/Chemokine Complex Building and CG-MD Simulations

**Figure 3:**
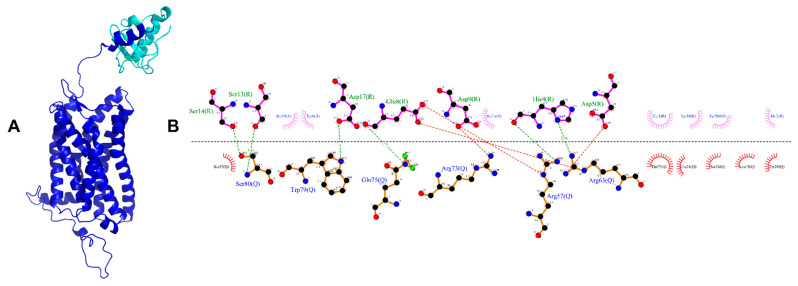
CXCR6‒CXCL16 molecular docking. (**A**) CXCR6‒CXCL16s complex; (**B**) interaction diagram, where R is CXCR6 and Q is CXCL16s; the hydrophobic environment is represented by residues in red and pink, the ionic interactions are represented by red dotted lines, and the H-bonds are represented by green dotted lines.

Using the minimized structure of CXCR6 and cluster1 of CXCL16s, protein–protein docking was carried out in the ClusPro server to observe the preliminarily protein‒protein interactions and to position the CXCR6‒CXCL16s complex for the CG-MD simulations. The docking was constrained by selecting the first 26 residues of CXCR6 as a binding site, based on the experimental mutagenesis data of other CXCRs (for example, CXCR3) [20]. Seventeen models of receptor–chemokine complex interactions were created by ClusPro on the combination of hydrophobic and electrostatic interactions (Figure S4). The conformation calculated by ClusPro that was chosen for the CG-MD simulations was the one that presented the highest number of conformations in the cluster based on the combination of hydrophobic and electrostatic interactions. The polar interactions between CXCR6 and CXCL16s of the best protein‒protein docking performed by ClusPro are presented in Figure 3.

In the first interactions between CXCL16s and CXCR6, acidic residues in CXCR6 like E8, D9, and D17 are interacting with basic residues in CXCL16s. The residue N16 in CXCR6 has one glycosylation (*N*-linked, GlcNAc), which was present in the minimization process but was removed during molecular docking because the ClusPro server did not recognize this post-translational modification. In the preliminary interactions, this residue is not present in polar interactions or a hydrophobic environment; the chemokine interacts with some residues and is positioned around 26 residues in restriction (Figure 3B). Once the complex was obtained, the system was built and a CG-MD 1-μs simulation was performed. Analysis (F0‒F10) was performed every 100 ns using Frame 0 (F0) as a control; this conformation was obtained after the minimization and equilibrium processes and the conversion to an all-atom model, to obtain rotation angles and polar interactions among TMs of the representative structure for each frame of the five runs of CG-MD simulations.

#### 3.3.1. CXCR6 CG-MD Simulations

Table 2 presents the residues of transmembrane segments obtained by UniProt (Code: O00574) [44] for the analysis of RMSD, RMSF, TM rotations, and polar interactions among TMs. 

**Figure 4:**
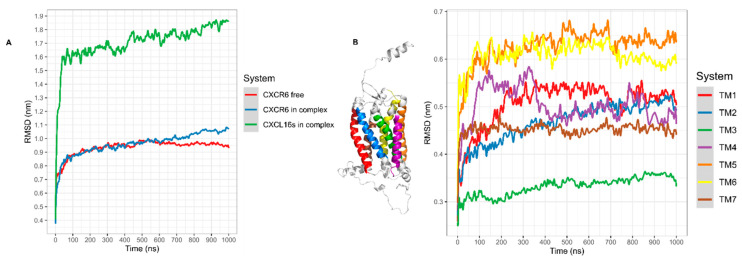
RMSD of CXCR6 CG-MD. (**A**) RMSD CXCR6, CXCR6 in complex with CXCL16s, and CXCL16s; (**B**) RMSD TMs.

In the CG-MD simulation, the CXCR6 GlcNAc was not added because CHARMM-GUI does not have the parameters of small molecules in the Martini Maker module. The system reaches equilibrium around 100 ns; greater changes occur before 100 ns, and this movement is related to system equilibration (Figure 4A, red line).

In the RMSD of TMs, all TMs showed greater movement before 100 ns; after this time, TM5 and TM6 showed the greater RMSD. However, these TMs maintained discrete movement throughout the simulation, with minor changes at 400 and 700 ns. Meanwhile, TM4 showed two relevant peaks in RMSD at 300 and 700 ns. TM3 was the TM with the least movement, increasing at 200 ns. TM2 increased the movement, although in the simulation TM1 had two relevant peaks in RMSD, the first at 100–300 ns, and after TM1 has a minor movement, the second peak occurred at 650–800 ns. TM7 showed less movement compared to TM1, TM2, TM4, TM5, and TM6, without relevant changes at the end of the simulation (Figure 4B). 

**Figure 5:**
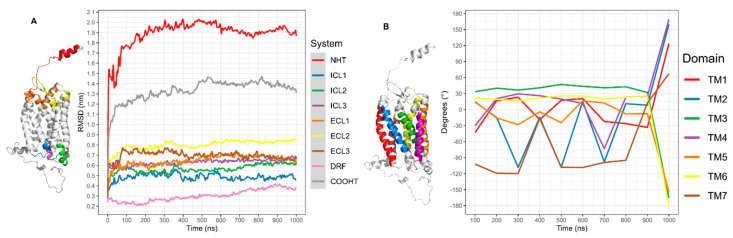
RMSD of CXCR6 CG-MD. (**A**) RMSD loops and (**B**) rotation of TMs.

In the case of extracellular and intracellular loops (ECLs and ICLs, respectively), both amino and carboxyl terminals exhibit great movement in comparison to extracellular and intracellular loops, as expected. ECL2 was the loop with the most movement and ICL1 was the loop with the least movement in the simulation. The DRF motif, which is an interesting point to analyze, showed a minor variation around 600–1000 ns (Figure 5A). In addition, the rotation of TMs was calculated to observe the behavior of TMs in the simulation (Figure 5B). TM1, TM2, and TM4 had the greatest rotation in the simulation, and at 600–700 ns TM1, TM2, TM4, and TM5 showed peaks of rotation. TM3 and TM6 were the TMs with the most minor rotations, and TM2 and TM4 showed continuous changes in the simulation.

The analysis of polar interactions between TMs was performed every 100 ns for the representative structure of the five runs (Table S1). Residue D77 (TM2) showed a constant interaction with residue R280 in TM7 in all frames of simulation (F0‒F10), whereas interactions between TM2 and TM3 were present in most frames of the simulation, mostly between A76, D77 (TM2), and N112 (TM3). TM3 and TM4 had a constant interaction in the simulation between T110 (TM3) and S163 (TM4), whereas TM3 interacted with TM6 between F113 (TM3)–Q244 (TM6) and S116 (TM3)–F240 (TM6). Meanwhile, TM5 and TM6 had interactions in all frames of the simulation; the residues involved were S188 (TM5), K251, R254, and S255 (TM6), Q195 (TM5)–Q244, and N248 (TM6). Moreover, TM6 had a constant interaction with TM7 between T243 (TM6) and R280 (TM7). 

The DRY motif is a sequence of three residues (aspartic acid, arginine, and tyrosine) conserved in Class A GPCRs and is relevant to the stabilization of active‒inactive states of GPCRs through an ionic interaction between aspartic acid and arginine called an “arginine cage”. Tyrosine (Y) interacts with TM6 in the meta-active state of rhodopsine, and once the arginine cage is broken, arginine (R) interacts with the alpha subunit of the G-protein [20,23,24]. However, in CXCR6 this motif is formed by aspartic acid, arginine, and phenylalanine—the DRF motif has been proposed as an explanation of how CXCR6 adapts to cellular adhesion when the receptor interacts with CXCL16m [22].

To observe the behavior of the DRF motif, and in an effort to elucidate its function, the polar interactions in each frame were observed. The “arginine cage” between D126 and R127 was broken at 800–1000 ns. The breaking of the arginine cage coincided with the decrease in TM2‒TM3 and TM3‒TM4 interactions. R127 had interactions before the arginine cage was broken between the sidechain and I123, Q225, Q141, and F224. At 800–1000 ns, the R127 sidechain interacted with Q141 and F224. The polar interactions in each frame of DRF are presented in Table S2.

#### 3.3.2. CXCR6‒CXCL16s CG-MD Simulations

**Figure 6:**
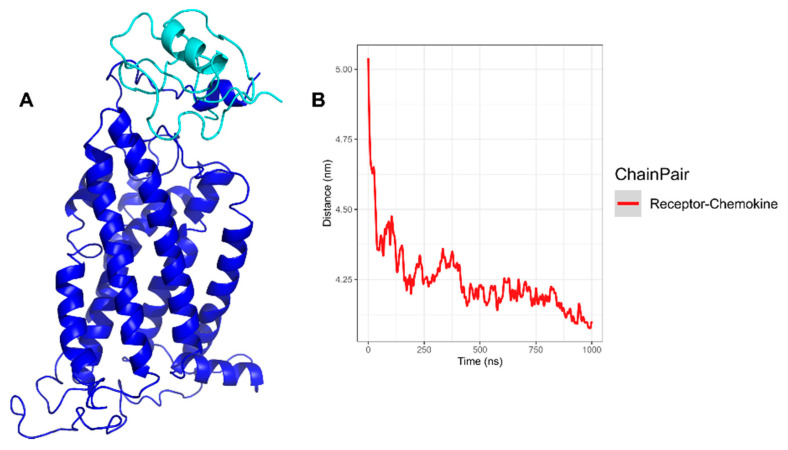
Distance between CXCR6‒CXCL16s CG-MD. (**A**) Structure of complex at 500 ns, with CXCL16s depicted in turquoise and CXCR6 in blue; (**B**) distance between center of mass of CXCL16s and CXCR6; we can observe a decrease in distance between CXCL16s and CXCR6, with an equilibrium around 500 ns.

In the conformations obtained from CG-MD for the CXCR6‒CXCL16s complex, the NH_2_-T was oriented to extracellular loops and the distance (center of mass) between CXCR6 and CXCL16s diminished over time, with an equilibrium in distance at 500–750 ns (Figure 6). Moreover, at the end of the simulation, the distance between the chemokine and the receptor decreased.

**Figure 7:**
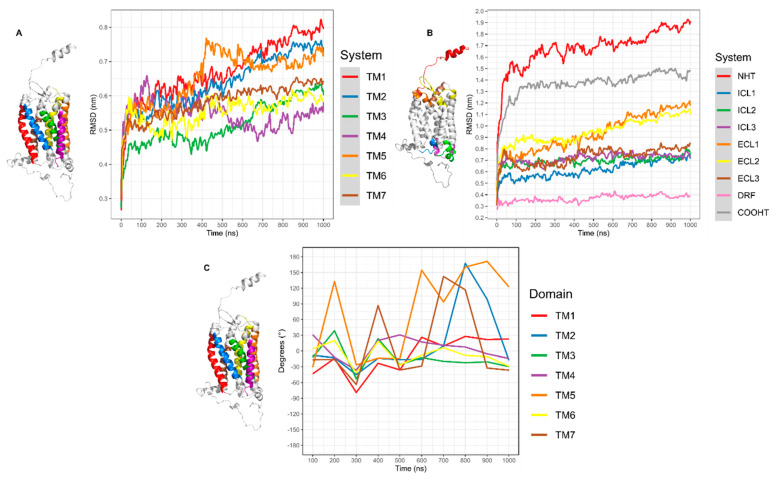
RMSD and rotation angles of CXCR6‒CXCL16s CG-MD simulation. (**A**) RMSD of system, CXCR6, and CXCL16; (**B**) RMSD of loops; (**C**) RMSD of TMs.

The RMSD of CXCR6 in complex with CXCL16s showed a moderate and continuous increase in RMSD; at 600 ns, the RMSD showed a mild increase until the end of the simulation (Figure 4A, blue line). CXCL16s presented the most drastic change in RMSD and showed a continuous increase in the simulation. The RMSD of the TMs showed different behavior in comparison to TMs without stimuli of CXCL16s. All TMs were in equilibrium before 400–500 ns; after this time TM1, TM2, TM3, and TM5 increased in movement. In contrast, TM4 decreased in movement and TM6 maintained discrete movement (Figure 7A). The RSMD of ECLs and ICLs indicated that ECL1 and ECL2 had greater movement with respect to ECL3 and ICLs. The DRF motif showed a discrete change in RMSD, although the simulation showed a mild increase at 400 ns. Furthermore, NH_2_-T showed continuous movement in the simulation. The maximum movement of NH_2_-T occurred toward the end of simulation (F10), and COOH-T remained stable at 100–1000 ns, with a small increase at 800 ns (Figure 7B). In addition, the angle of rotation of TMs was variable, with TM5 being the transmembrane segment with the greatest rotation. At 600 ns, TM1, TM2, TM5, and TM7 had a greater peak in rotation; meanwhile, TM3, TM4, and TM6 decreased their rotation. Before 600 ns, all TMs had variations in their rotation (Figure 7C).

In the chemokine‒receptor interaction, a “two-site” model for complex formation has been proposed, in which the receptor recognizes the site localized in the docking domain (*N*-loop region) after the two first cysteines [85], facilitates the positioning, and increases interactions between CXCL16s and CXCR6, inducing conformational changes.

The residues involved in the interactions between CXCL16s and CXCR6 (Table S3) were acidic residues in CXCR6 (aspartic and glutamic acid) and basic residues in CXCL16 (lysine and arginine). The interaction between E259 (CXCR6) and R43 (CXCL16s) was present in all frames. The residues R63, R67, and R73 in CXCL16s showed an interaction with acidic residues in CXCR6 through the 1-μs CG simulation.

The polar interactions between TMs are presented in Table S4. TM1 had a few interactions, whereas only TM7 showed one between N49 and P285. TM2 interacted constantly with TM7; the polar interaction between D77 and R280 was present throughout the simulation, and interactions with TM3 decreased in the presence of CXCL16s. TM3 had a constant interaction with Q244 in TM6, and TM4 had a few interactions with TM2 and TM3. TM5, however, lost interactions with TM6, whereas interactions between Q195, Q244, and N248 were lost in the presence of CXCL16s. This interaction was, however, present in the CXCR6 CG-MD and could be relevant to stabilizing the inactive state of CXCR6. TM6 interacted constantly with TM7 between T243 and R280.

The changes in the polar interactions of TMs could be attributed to CXCL16s’ contact with the receptor, which breaks the interactions between TM5 and TM6. Additionally, the arginine cage is broken at 500 ns and the R127 sidechain interacts with I65, T66, Y138, Y288, and S292.

Contrasting the RMSF of TMs and ICL2 (containing the DRF motif), some relevant residues increased or decreased their general movement. Although the 1 μs simulation included stimuli of CXCL16s (Figures S5 and S6), the most representative changes were that Y109 in TM3 decreased the RMSF value by approximately 0.15 nm, and N112 and F113 by approximately 0.05 nm. In contrast, F194 and Q195 increased the RMSF value by approximately 0.05 nm, whereas M196, T197, L198, G199, and F200 increased it by approximately 0.1 nm. Furthermore, the DRF motif presented a mild decrease in movement, and R127 and F128 were the residues with major RMSF values (Figures S5H and S6H).

The CG-MD simulation allowed us to observe the two steps involved in chemokine receptor activation: (1) the interaction and decreased distance between CXCL16s and CXCR6, and (2) the conformational changes and rotation of the transmembrane segments in CXCR6, derived from the chemokine‒receptor interaction [17,18,19]. TM2‒TM3, TM2‒TM4, TM2‒TM7, TM3‒TM6, TM5‒TM6, and TM6‒TM7 interactions were present in both simulations. When the chemokine was present, the interactions between TM2‒TM4 decreased, and the interactions of TM5‒TM6 with Q195‒Q244 and N248 were lost in the presence of CXCL16s. The interactions between D77‒R280 and T243‒TM7 was able to be related to the functional conformation of CXCR6; alternatively, the interaction between Q195‒Q244 and N248 was able to be related to the inactive state, and the loss of this interaction and the arginine cage breaking in the presence of CXCL16s allowed for the meta-active state of CXCR6.

### 3.4. General Protein‒Ligand Interaction Model and Receptor-Based 3D-QSAR Model of CXCR6 Antagonists 

To support the design of new antagonists to CXCR6, a general protein–ligand interaction model was built, based on 82 ligands reported to be antagonists for CXCR6 [36]. The general protein‒ligand interaction model is a representation of the pharmacophoric features important for the recognition of a ligand by CXCR6. The representative structure of F0 of CXCR6 CG-MD was used for molecular docking, using this conformation to design new molecules that can antagonize CXCR6 before chemokine stimulation in order to avoid chemotaxis of cells. Once the molecular docking complexes were obtained, the interactions present were grouped for the construction of a general protein–ligand interaction model to observe the pharmacophoric features in a 3D position, which are relevant for the interaction of antagonists with CXCR6, considering the pIC50 of molecules (Table S6). The molecular docking of the 82 molecules reported as antagonists of CXCR6 was performed, with the docking scores (DS) shown in Table S6. The 2D structure indicated the three best and three worst ligands in pIC50, as shown in Figure S7. Ligand 75 had the best docking score (−10.74 kcal/mol), whereas ligand 82 had the best pIC50 (7.40) and a docking score of −8.92 kcal/mol.

#### 3.4.1. General Protein‒Ligand Interaction Model Based on pIC50

**Figure 8:**
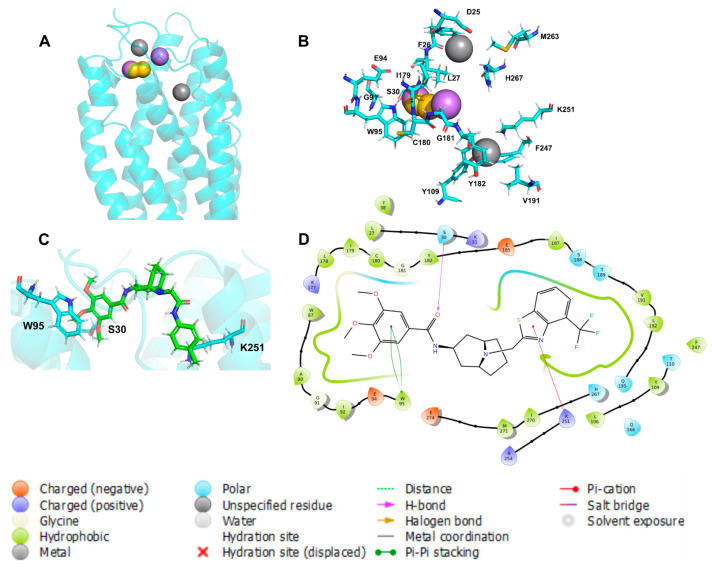
General protein–ligand interaction model based on pIC50. (**A**) General model: hydrogen bond acceptors (HAc) depicted in magenta, hydrogen bond donors (HDn) in gray, and hydrophobic groups (Hph) in yellow. (**B**) Residues around 5 Å; (**C**) 3D conformation of ligand 82; (**D**) ligand interaction diagram of ligand 82.

The general model based on pIC50 presented three relevant pharmacophoric features, HAc, HDn, and Hph, distributed around the space between ECLs and TMs (Figure 8A,B); the residues around 5 Å of chemical features are presented in Table 3, and the coordinates of the model are presented in Table S7.

The HDn feature is close to Y109 and K251 in TM3 and TM6; these transmembrane segments are involved in the functional conformation of CXCR6, blocking the rotation or polar interaction of transmembrane segments that could destabilize the receptor and block their function. In contrast, HAc and Hph features are close to ECL1 and ECL2 and could be blocking the interactions of CXCL16s and the ECLs.

Ligand 82 interacts with W95, a residue present in the general model, and possibly acts as an anchor to electron-rich aromatic rings, such as the 2,3,4-trimethoxyphenyl moiety, for the binding of the antagonist to CXCR6. By blocking the movement and interaction of TM3, TM6 molecules could antagonize the CXCR6 receptor. The position of ligand 82 is near to Q195 and E274 (Figure 8D), key residues in CXCL16s’ binding to the inactive state of the receptor.

These data support the proposal that the interaction between Q195 in TM5 and Q244 and N248 in TM6 is key to CXCR6′s inactive and functional state. New molecules with those pharmacophoric features would bind to CXCR6 and antagonize the receptor.

#### 3.4.2. Receptor-Based 3D-QSAR Model

In addition to the information provided for the general protein‒ligand interaction model, a 3D-QSAR analysis was performed to verify the pharmacophoric features related to the activity of the 82 molecules. Ten models were built, and the statistical parameters considered to select the three best models were *q*^2^ (>0.5), *R*^2^ (≥0.85), and the F-test values calculated by OPEN3DQSAR. The VdW and electrostatic fields were calculated for all molecules; the test group consisted of 23 randomly selected molecules. Table 4 presents a summary of the statistical values and ligands of the test group of the best models. Statistics on the other models are presented in Table S7. 

**Figure 9:**
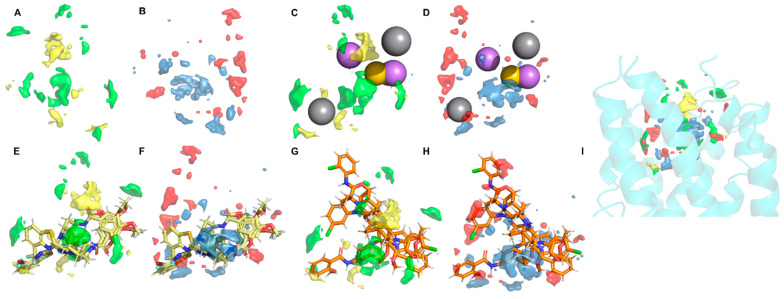
3D-QSAR model. (**A**) Pharmacophoric features in the QSAR model (in yellow: negative VdW field; in green: positive VdW field); (**B**) pharmacophoric features in the QSAR model (in red: negative electrostatic field; in light blue: positive electrostatic field); (**C**) superposition of general protein‒ligand interaction model and VdW fields; (**D**) superposition of general protein‒ligand interaction model and electrostatic fields; (**E**) the three ligands with the best pIC50 (82, 81, and 78) in VdW fields; (**F**) the three ligands with the best pIC50 in electrostatic fields; (**G**) the three ligands with the worst pIC50 (51, 56, and 55) in VdW fields; (**H**) the three ligands with the worst pIC50 in electrostatic fields; (**I**) the three-dimensional structure of the QSAR model.

The dependent variable used in the 3D-QSAR calculations was pIC50, and model 7 was the best model, with a *R*^2^ value higher than 0.8. This model was used to represent the principal chemical features of the interaction with CXCR6 and to support the information provided by the general protein‒ligand interaction models to associate the pharmacophoric features with the ability to bind and antagonize the CXCR6 receptor (Figure 9).

The positive steric (VdW), and electrostatic fields coincide with the steric and positive electrostatic region of the *exo*-azabicyclo[3.3.1]nonane scaffold in the best pIC50 ligands, being close to the E274 residue relevant to the activity of CXCR6. The 2,3,4-trimethoxyphenyl ring acts as an anchor for binding, and the QSAR model indicates that the zone requires electron-rich and steric scaffolds. Once the ligand interacts in this zone, the orientation of the model coincides with the general model based on pIC50. The pharmacophoric features of Hph, HAc, and HDn are necessary for binding and activity: HAc provides the negative electrostatic field and Hph provides the positive steric field in the region of the 2,3,4-trimethoxyphenyl ring. Overlapping with the 3D-QSAR model in the CXCR6 structure, the QSAR model is oriented to ECLs and TMs. This information supports our results in molecular dynamics: the interruption in TM3‒TM5‒TM6 interactions disabled the ability of CXCR6 to adopt a meta-active conformation.

## 4. Conclusions

The CXCR6‒CXCL16 axis is relevant to several pathologies; thus, the inhibition of this axis is an important research hotspot in the rational design of new molecules. In this work we present a homology model of CXCR6 and CXCL16s; both models, after minimization processes, are useful to observe the interactions and behavior of CXCR6. In the CG-MD simulations, the NH_2_-T in complex with CXCL16s is positioned in the middle of ECLs oriented to TMs and triggers the system’s evolution, rotations, movements, and TM interactions, changing the inactive state to a meta-active state.

The interaction of D176 (CXCR6) and residues in CXCL16s present in frames of the simulation is in agreement with the reported experimental data [15]; other residues involved in this interaction are acidic residues in CXCR6 and basic residues in CXCL16s. The interaction between E259 and R43 is present in all frames of the simulations and might suggest that the E259 residue is relevant to the binding of CXCL16s.

The CG-MD simulations reveal movements and interactions related to the receptor’s inactive or active states. The network of polar interactions between TM3, TM5, and TM6 without the chemokines as stimuli and the interactions between TM2 and TM7 showed that they are involved in the inactive state and the functional conformation of CXCR6. When CXCL16s interacts and orients itself towards the TMs, the rotation of TM5 and TM6 destabilizes the interaction between them and the interactions between Q195, Q244, and N248 are lost. This type of interaction is a research hotspot in the rational design of antagonists of CXCR6 to destabilize the functional conformation of CXCR6. When the chemokine is present, the two-step activation is observed—the chemokine interacts with NH_2_-T and is oriented to TMs, and conformal changes are observed.

The general protein‒ligand interaction model allowed us to observe the pharmacophoric features necessary to bind and antagonize the CXCR6 receptor, in which HAc, HDn, and Hph are relevant to the ligand’s activity and bind in the zone with a higher ability to break the TM3‒TM5‒TM6 polar network. The proximity of ligand 82 (pIC50 = 7.398) to Q195 supports the results provided by the CG-MD simulations: residue W95, located in ECL2, acts like an “anchor” to aromatic rings in the evaluated ligands, and the orientation to TM3 and TM6 is fundamental to antagonize CXCR6. This information can be used in the rational design of new molecules that can prevent the binding of CXCL16s to CXCR6, in the orientation towards the transmembrane region, or to break the net of the polar interaction of CXCR6 (TM3‒TM5‒TM6), avoiding the conformational change into a meta-active state of the receptor and, consequently, causing a reduction of chemotaxis in pathologies in which this axis is involved.

## Figures and Tables

**Table 1:** Summary of quality of structures.

ID	C-Score	TM-Score	Ramachandran Favored	* z-Score
CXCR6-M1	−0.46	0.65 ± 0.13	85.29%	−3.03 ± 0.38
CXCR6	-	-	84.71%	−3.08 ± 0.37
CXCR6 **	-	-	91.76%	−2.05 ± 0.39
CXCL16s-M1	−1.51	0.53 ± 0.15	67.57%	−6.24 ± 0.71
CXCL16s-C1	-	-	89.19%	−2.50 ± 0.81

* Z-score: between −3 and 3 is a good conformation [84], ** minimized structure.

**Table 2:** Residues of transmembrane segments in CXCR6.

TM1	TM2	TM3 *	TM4	TM5	TM6	TM7
33–59	69–89	104–128	144–164	188–215	232–259	276–293

* The residues of DRF are included (126‒128).

**Table 3:** Pharmacophoric features of general model based on pIC50.

Pharmacophoric Features	Residues Around 5 Å
HAc	**L27**, **S30**, G91, E94, **W95**, **I179**, **C180**, G181, **Y182**
HDn	D25, F26, **L27**, Y109, **Y182**, V191, F247, K251, M263, H267
Hph	**L27**, **S30**, **W95**, **I179**, **C180**

In bold are the residues that are repeated in the different pharmacophoric features.

**Table 4:** Statistical values of QSAR models.

Models	q^2^	*R^2^*	F Test	Test Group
Model 7	0.6397	0.8731	72.8899	18, 74, 38, 2, 72, 80, 28, 52, 16, 15, 12, 73, 70, 26, 47, 13, 32, 54, 65, 62, 59, 79, 56
Model 1	0.6329	0.8639	67.2740	33, 30, 39, 22, 61, 28, 36, 53, 9, 66, 56, 59, 24, 48, 72, 18, 2, 52, 13, 41, 62, 46, 75
Model 4	0.4906	0.8718	72.0680	27, 50, 25, 9, 2, 23, 14, 67, 41, 70, 33, 28, 39, 79, 66, 36, 13, 1, 71, 55, 82, 3, 76

## Data Availability

The data presented in this study are available in Supplementary Material.

## Supplementary Materials

The following are available online at https://www.mdpi.com/article/10.3390/life11040346/s1, Figure S1: Ramachandran plots; Figure S2: Sequence alignment by BLAST; Figure S3: Alignment of CXCR6 and CXCL16s structures; Figure S4: First ten models obtained in ClusPro server; Figure S5: RMSF of CXCR6 CG-MD; Figure S6: RMSF of CXCR6-CXCL16s CG-MD; Figure S7: 2D structure of three best and three worst ligands; Table S1: Polar interactions between transmembrane segments in CXCR6 CG-MD; Table S2: Polar interactions of DRF motif in CXCR6 CG-MD; Table S3: Residues involved in the interactions between CXCR6-CXCL16s CG-MD; Table S4: Polar interactions between transmembrane segments in CXCR6-CXCL16 CG-MD; Table S5: Polar interactions of DRF motif in CXCR6-CXCL16 CG-MD; Table S6: Docking scores and pIC50 of 82 antagonist of CXCR6; Table S7: General protein-ligand nteraction model coordinates; Table S8: Statistical values of QSAR models.
