# Supplementary material for: Behavior of Chemokine Receptor 6 (CXCR6) in Complex with CXCL16 Soluble form Chemokine by Molecular Dynamic Simulations: General Protein‒Ligand Interaction Model and 3D-QSAR Studies of Synthetic Antagonists
Source: Life (Basel). 2021 Apr 15;11(4):346. doi: 10.3390/life11040346 (PMC8071165; doi:10.3390/life11040346)
Supplement: Supplementary file 1 [file life-11-00346-s001.zip › life-1189143-supplementary.pdf]

Supplementary Materials

Behavior of Chemokine Receptor 6 (CXCR6) in Complex with CXCL16 Soluble form Chemokine by Molecular Dynamic Simulations: General Protein–Ligand Interaction Model and 3D-QSAR Studies of Synthetic Antagonists

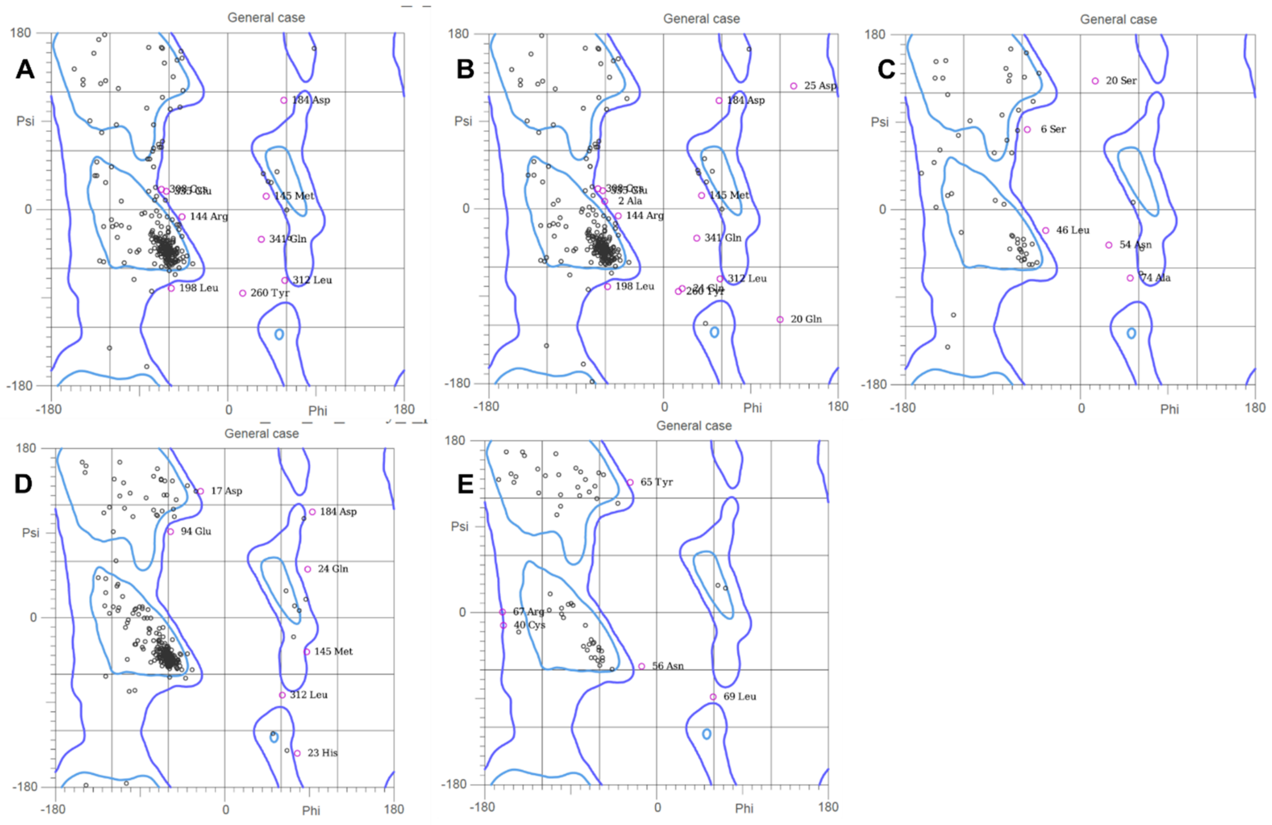

Figure S1. Ramachandran plots. A) CXCR6-M1, B) CXCR6-reoriented, C) CXCL16s, D) CXCR6-minimizated, E) CXCL16s cluster1.

```
A Score             Expect  Method                          Identities      Positives       Gaps
  175 bits(444)     1e-56   Compositional matrix adjust.    107/308(35%)    171/308(55%)    18/308(5%)

Query  2    DYQVSSPIYDINYYTSEPCQKINVKQIAARLLPPLYSLVFIFGFVGNMLVILILINCKRL  61
            DY         N  + E  Q  +  Q +   LP +Y +VF+ G VGN LV++I I   +L
Sbjct  5    DYHEDYGFSSFNDSSQEEHQ--DFLQFSKVFLPCMYLVVFVCGLVGNSLVLVISIFYHKL  62

Query  62   KSMTDIYLLNLAISDLFFLLTVPFWAHYAAAQWDFGNTMCQLLTGLYFIGFFSGIFFIIL  121
            +S+TD++L+NL ++DL F+ T+PFWA+    +W FG  MC+ L G+Y I F++ +  +
Sbjct  63   QSLTDVFLVNLPLADLVFVCTLPFWAYAGIHEWVFGQVMCKSLLGIYTINFYTSMLILTC  122

Query  122  LTIDRYLAVVHAVFAL--KARTVTFGVVTSVITWVVAVFASLPGIIFTRSQKEGLHYTCS  179
            +T+DR++ VV A  A   +A+ +T+G VTS++ WV+++   SLP II+          C
Sbjct  123  ITVDRFIVVVKATKAYNQQAKRMTWGKVTSLLIWVISLLVSLPQIIYGNVFNLD-KLICG  181

Query  180  SHFPYSQYQFWKNFQTLKIVILGLVLPLLVMVICYSGILKTLLRCRNEKKRHRAVRLIFT  239
             H             T     LG  LPLL M++CYS I+KTLL     +K HR++++IF
Sbjct  182  YHDEAISTVVLATQMT-----LGFFLPLLTMIVCYSVIIKTLLHAGGFQK-HRSLKIIFL  235

Query  240  IMIVYFLFWAPYNIV-LLLNTFQEFFGLNNCSSSNRLDQAMQVTETLGMTHCCINPIIYA  298
            +M V+ L    P+N++  + +T  E++ + +           + VTE +     C+NP++YA
Sbjct  236  VMAVFLLTQMPFNLMKFIRSTHWEYYAMTS------FHYTIMVTEAIAYLRACLNPVLYA  289

Query  299  FVGEKFRN  306
            FV  KFR
Sbjct  290  FVSLKFRK  297

B Score             Expect  Method                          Identities      Positives       Gaps
  20.0 bits(40)     0.001   Compositional matrix adjust.    10/32(31%)      18/32(56%)      3/32(9%)

Query  37   IFHTKKKL---SVCANPKQTWVKYIVRLLSKK  65
            +++T+ +L   SVC   K  WV+ ++  L  K
Sbjct  38   LYYTRFQLLSWSVCGGNKDPWVQELMSCLDLK  69
```

Figure S2. Sequence alignment by BLAST A) Alignment between CCR5 and CXCR6 and B) Alignment between CCL20 and CXCL16s.

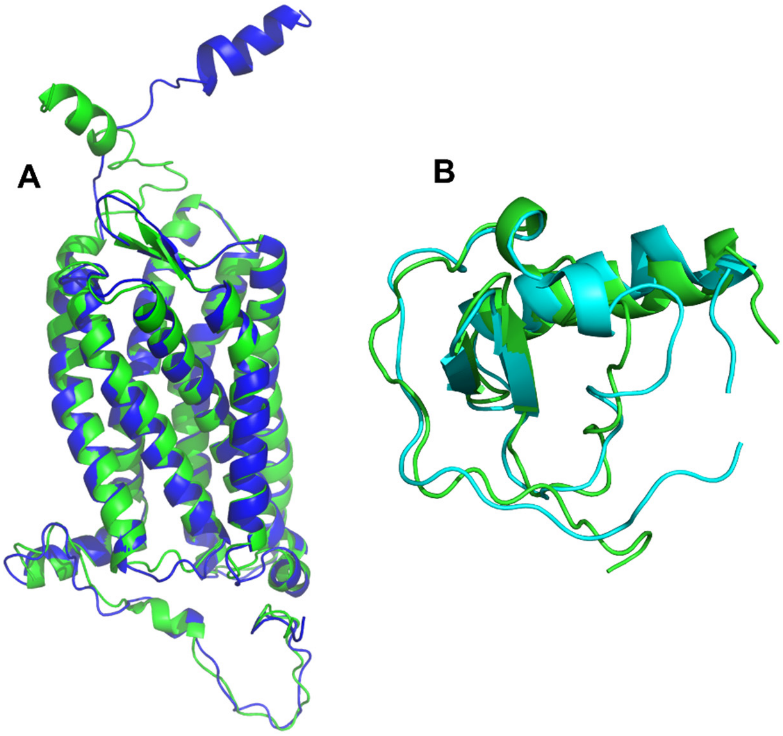

Figure 3. Alignment of CXCR6 and CXCL16s structures. A) CXCR6-M1 (green) and CXCR6-minimized (Blue), B) CXCL16s-M1 (green) and CXCL16s-C1 (turquoise).

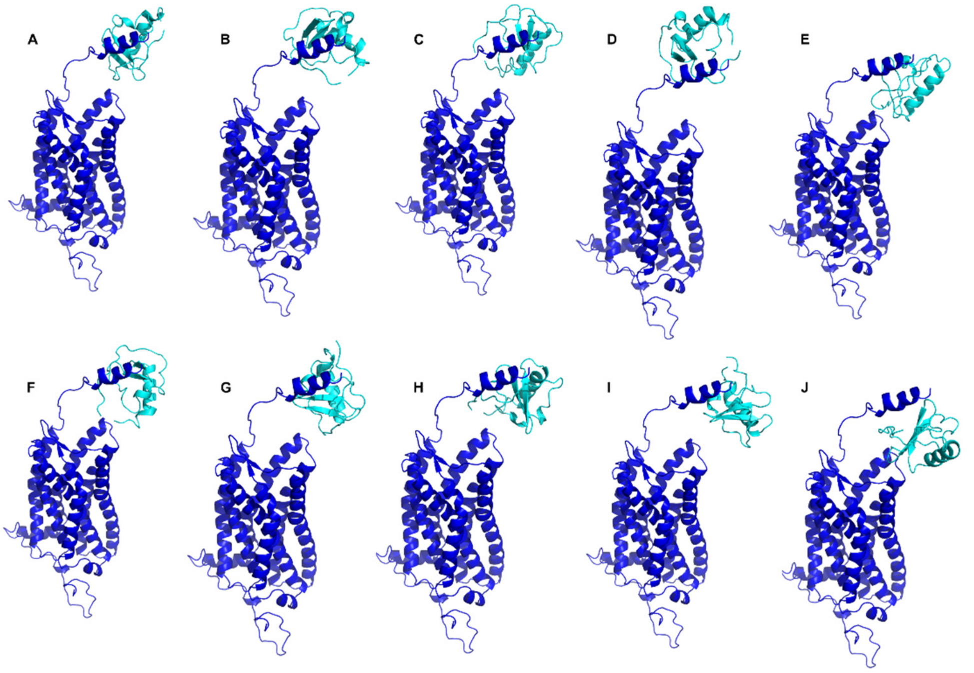

Figure 4. First ten models obtained in ClusPro server. A) model0, B) model1, C) model2, D) model3, E) model4, F) model5, G) model6, H) model7, I) model8, and J) model9. The model 0 was used to subsequent simulations.

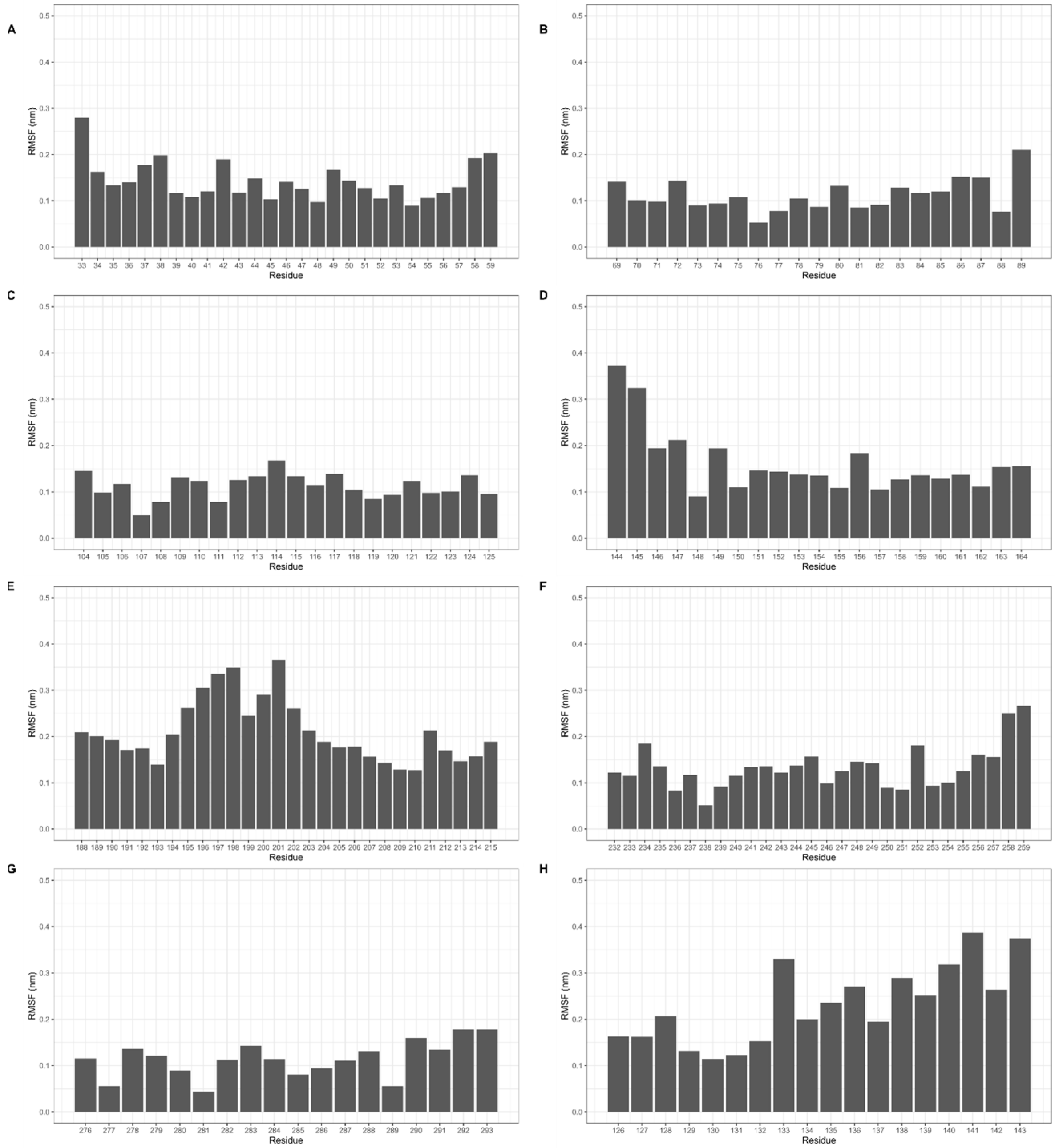

Figure S5. RMSF of CXCR6 CG-MD. A) TM1, B) TM2, C) TM3, D) TM4, E) TM5, F) TM6, G) TM7, H) ICL2.

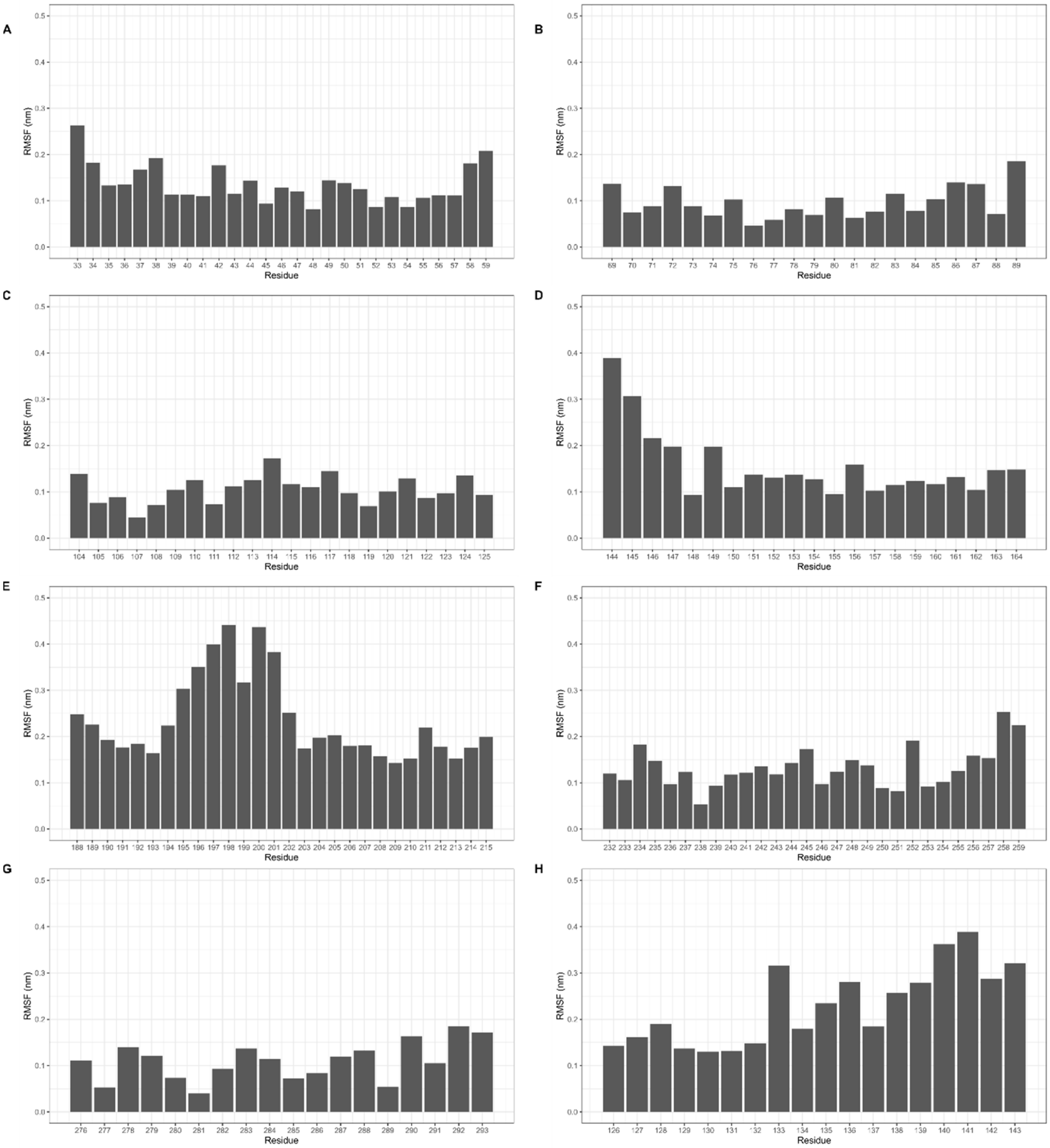

Figure S6. RMSF of CXCR6-CXCL16s CG-MD. A) TM1, B) TM2, C) TM3, D) TM4, E) TM5, F) TM6, G) TM7, H) ICL2.

Table S1. Polar interactions between transmembrane segments in CXCR6 CG-MD.

0 ns	TM1	TM2	TM3	TM4	TM5	TM6	TM7
TM1	-	-	-	-	-	-	-
TM2	-	-	-	N72-S152	-	-	D77-R280, N284
TM3	-	-	-	I111-S159	Y114-T194	-	S116-R280
TM4	-	-	-	-	-	-	-
TM5	-	-	-	-	-	Q195-Q244	-
TM6	-	-	-	-	-	-	-

100 ns	TM1	TM2	TM3	TM4	TM5	TM6	TM7
TM1	-	-	-	-	-	-	N79-R285
TM2	-	-	A76-N112	N72-W156	-	-	A76, D77-R280
TM3	-	-	-	-	T110-V162	T124-T206	N112-R280
TM4	-	-	-	-	-	-	-
TM5	-	-	-	-	-	Q195-N248<br>G199-Q244	-
TM6	-	-	-	-	-	-	T243-R280

200 ns	TM1	TM2	TM3	TM4	TM5	TM6	TM7
TM1	-	-	-	-	-	-	-
TM2	-	-	D77-N112	-	-	-	D77-R280
TM3	-	-	-	T110-S163<br>I111-S159	-	-	-
TM4	-	-	-	-	-	-	-
TM5	-	-	-	-	-	S188-k251, S255<br>Q195-Q244	-
TM6	-	-	-	-	-	-	T243-R280

300 ns	TM1	TM2	TM3	TM4	TM5	TM6	TM7
TM1	-	-	-	-	-	-	-
TM2	-	-	A77-T115	-	-	-	D77-R280
TM3	-	-	-	-	-	S116-F241	Y109-Y278<br>N112-R280
TM4	-	-	-	-	-	-	-
TM5	-	-	-	-	-	S188-K251, R254	-
TM6	-	-	-	-	-	-	T243-R280

400 ns	TM1	TM2	TM3	TM4	TM5	TM6	TM7
TM1	-	-	-	-	-	-	N49-P285
TM2	-	-	D77. F80-N112<br>W87-Y109	-	-	-	D77-R280
TM3	-	-	-	T110-S163<br>I111-S159	-	S116-F240	N112-R280
TM4	-	-	-	-	-	-	-
TM5	-	-	-	-	-	S188-K251<br>Q195-N248	-
TM6	-	-	-	-	-	-	-

500 ns	TM 1	TM 2	TM3	TM4	TM5	TM6	TM7
TM1	-	-	-	-	-	-	-
TM2	-	-	A76-N112, T115<br>D77-N112<br>W87-Y109	-	-	-	D77-R280, N284
TM3	-	-	-	T110-S163	T110-Q195	-	-
TM4	-	-	-	-	-	-	-
TM5	-	-	-	-	-	S188-R254, S255<br>Q195-Q244	-
TM6	-	-	-	-	-	-	-

600 ns	TM1	TM2	TM3	TM4	TM5	TM6	TM7
TM1	-	-	-	-	-	-	N49-P285
TM2	-	-	-	-	-	-	D77-R280
TM3	-	-	-	T110-S163	-	S116-F240	Y109-Y278<br>N112-R280
TM4	-	-	-	-	-	-	-
TM5	-	-	-	-	-	S188-R254<br>Q195-Q244, N248<br>Y211-F234	-
TM6	-	-	-	-	-	-	-

700 ns	TM 1	TM 2	TM3	TM4	TM5	TM6	TM7
TM1	-	-	-	-	-	-	-
TM2	-	-	A76, D77-N112	-	-	-	D77-R280, N284

TM3	-	-	-	L106, T110-<br>S163<br>T110-S159	-	F113-Q244	-
TM4	-	-	-	-	S159-<br>Q195	-	-
TM5	-	-	-	-	-	S188-S255<br>Q195-Q244,<br>N2480	-
TM6	-	-	-	-	-	-	T243-R280

**800 ns**	**TM1**	**TM2**	**TM3**	**TM4**	**TM5**	**TM6**	**TM7**
TM1	-	-	-	-	-	-	N49-P285
TM2	-	-	-	-	-	-	D77-R280
TM3	-	-	-	-	Y114-Q194	F113-Q245	N112-R280
TM4	-	-	-	-	-	-	-
TM5	-	-	-	-	-	S188-R254<br>Q195-N248	-
TM6	-	-	-	-	-	-	V236-N284

**900 ns**	**TM1**	**TM2**	**TM3**	**TM4**	**TM5**	**TM6**	**TM7**
TM1	-	-	-	-	-	-	N49-P285
TM2	-	-	-	N72-S152	-	-	D77-R280, N284
TM3	-	-	-	-	Y114-T194<br>T124-T206	S116-Q244	-
TM4	-	-	-	-	-	-	-
TM5	-	-	-	-	-	Q195-N248<br>Y211-F234	-
TM6	-	-	-	-	-	-	-

**1000 ns**	**TM1**	**TM2**	**TM3**	**TM4**	**TM5**	**TM6**	**TM7**
TM1	-	-	-	-	-	-	-
TM2	-	-	-	-	-	-	D77-R280, 284
TM3	-	-	-	-	-	-	-
TM4	-	-	-	-	-	-	-
TM5	-	-	-	-	-	-	-
TM6	-	-	-	-	-	-	F240-R280

Table S2. Polar interactions of DRF motif in CXCR6 CG-MD.

**0 ns**		**100**	**200**	**300**	**400**	**500**	**600**	**700**	**800**	**900**	**1000**
**D126**	C122	*Q141, C122, I129	C122, I129, V130	*K143, C122, V130	*K143, C122	*K143, C122, I129, V130	*K143, C122, I129, V130	*K143, C122	*Y138, *K143, C122	C122, V131, Q140, Q141	*Q140, *K143, C122, V130
**R127**	T124	I123, T124	T124	*I123, T124, V131	I124	*Q225, I123	I123	*Q141, *F224, I123	*Q141, I123	*Q141, *F224	*Q141, I123, T124
**F128**	T124	T1T12 4, V132	T124	T124, V131	V125	V125, V132	V125	T124, V125	T124, V125	T124	T124, V131

*Interactions between sidechain of DRF residue. Residues non-polar interacts although mainchain.

Table S3. Residues involved in the interactions between CXCR6-CXCL16s CG-MD.

0 ns		100 ns		200 ns		300 ns		400 ns		500 ns		600 ns		700 ns		800 ns		900 ns		1000 ns	
**R**	**Q**	**R**	**Q**	**R**	**Q**	**R**	**Q**	**R**	**Q**	**R**	**Q**	**R**	**Q**	**R**	**Q**	**R**	**Q**	**R**	**Q**	**R**	**Q**
E259	R43	D5	R67, Q75	E259	R43, T72, K86	D5	R67	Y260	R43	D25	K42, R57	E259	R43	W258	S33	E259	R43	E21	L77	E259	R43
S18	S78	D9	S37, R67, R73, Q75	D25	R43, I44	M1	R63	E259	R43, G83	E8	K42, R57	D17	K42	E3	R59	D17	K42	D17	K42, S46	E185	G36
D17	K42	G11	T35	E22	K42	E3	R63	E21	R73	Y6	T35, G36	E185	S33	E259	*R43	E3	R59	E259	R43	E21	R57
D9	R67, R73, Q75	H7	S33	E21	S37	G11	S37	S13	R67, R73	G11	T35	D5	R73	Y260	G41	H4	R59	D9	R67	D184	R67
E8	R57, *R63, Q75	E259	*R43, S46	S255	S33	D25	K42	D5	*R57, R59	D9	R67, R73, S80	E8	R63, *R73	D9	R67	D5	R73	E3	R59	D9	R73
S13	R73, Q75	S13	R43	N16	S37, R73	E21	R43	E8	R57, H61	D5	R67	E3	R59	D17	*K42	E8	R63, *R73	E8	R63	D5	R73
M1	T35	S14	R43	S14	S45, R73	S18	R43	D9	R67, Q75	S14	R43	S14	R67	E8	R63, A64, R73	D9	R67	D75	R73	E8	R63, R73
D5	T35, G36	Q24	N56	S13	S45, R73, S80	E22	*R43	H4	*D47, R57	N16	R43	D9	*H66, R67	D5	R73	Y10	C38			H4	H61
		E22	K42	H7	*D47, R57	Y260	K42	H7	D47	E259	R43			S19	S80	D184	R67			N16	K42, L77
		E8	K42, R57	E8	R57, R59	E259	S45	Q20	S33	E3	R63			D184	S37					Y268	K42
		E3	R63	D5	R59	N16	R43, S45	H183	S33	D176	G32			E21	S78					E3	R59
		H4	H61	D9	R67, S78	H4	S78	S19	G32	T264	S51										

E8	R57, *H61, W79	Y261	S48
H7	S33, R57		
Y10	G41		
D9	G41, R73		
Y6	G36		
D176	G32, T35		
K177	G32		

*Salt bridges interactions.

Table S4. Polar interactions between transmembrane segments in CXCR6-CXCL16 CG-MD.

**0 ns**	**TM1**	**TM2**	**TM3**	**TM4**	**TM5**	**TM6**	**TM7**
TM1	-	-	-	-	-	-	-
TM2	-	-	-	-	-	-	D77-R280<br>T83-Y278
TM3	-	-	-	G107, T110-S163	-	Y110-K251	-
TM4	-	-	-	-	-	-	-
TM5	-	-	-	-	-	L192, Q195-N248	-
TM6	-	-	-	-	-	-	-

**100 ns**	**TM1**	**TM2**	**TM3**	**TM4**	**TM5**	**TM6**	**TM7**
TM1	-	-	-	-	-	-	N49-O285
TM2	-	-	-	N72-S152	-	-	D77-R280, N284
TM3	-	-	-	T110-S163	Y114-T194	F133-Q244	-
TM4	-	-	-	-	-	-	-
TM5	-	-	-	-	-	-	-
TM6	-	-	-	-	-	-	T243, Q244-280

**200 ns**	**TM1**	**TM2**	**TM3**	**TM4**	**TM5**	**TM6**	**TM7**
TM1	-	N49-D77	-	-	-	-	-
TM2	-	-	A76, D77-N112	-	-	-	D77-R280
TM3	-	-	-	-	-	N112-Q244	N112-R280
TM4	-	-	-	-	-	-	-
TM5	-	-	-	-	-	-	-
TM6	-	-	-	-	-	-	V236-N284<br>T243-R280

**300 ns**	**TM1**	**TM2**	**TM3**	**TM4**	**TM5**	**TM6**	**TM7**
**TM1**	**-**	**-**	**-**	**-**	**-**	**-**	**N49-P285**
**TM2**	**-**	**-**	**-**	**-**	**-**	**-**	**D77-R280, N284**
**TM3**	**-**	**-**	**-**	**-**	**Y114-T197**	**Y109-K251**<br>**S116-Q244**	**-**
**TM4**	**-**	**-**	**-**	**-**	**-**	**-**	**-**
**TM5**	**-**	**-**	**-**	**-**	**-**	**Q195, F200-N248**<br>**Y211-F234**	**-**
**TM6**	**-**	**-**	**-**	**-**	**-**	**-**	**T243-R280**

400 ns	TM1	TM2	TM3	TM4	TM5	TM6	TM7
TM1	-	-	-	-	-	-	-
TM2	-	-	W87-Y109	-	-	-	D77-R280
TM3	-	-	-	-	-	N112-Q244<br>S116-F240	-
TM4	-	-	-	-	-	-	-
TM5	-	-	-	-	-	-	-
TM6	-	-	-	-	-	-	T243-R280

500 ns	TM1	TM2	TM3	TM4	TM5	TM6	TM7
TM1	-	-	-	-	-	-	Y38-Y278<br>N49-P285
TM2	-	-	T83-N112	N72-S152	-	-	D77-N284
TM3	-	-	-	G107, T110-S163	-	-	-
TM4	-	-	-	-	-	-	-
TM5	-	-	-	-	-	G199-Q244	-
TM6	-	-	-	-	-	-	Q244-R280

600 ns	TM 1	TM2	TM3	TM4	TM5	TM6	TM7
TM1	-	S56-L70	-	-	-	-	-
TM2	-	-	A76-S116	N72-S152	-	-	D77-R280
TM3	-	-	-	T115-S159	T110-Q195<br>M117, T121-T206	M117, F113-Q244	-
TM4	-	-	-	-	-	-	-
TM5	-	-	-	-	-	S188-R254	-
TM6	-	-	-	-	-	-	T243-R280

700 ns	TM1	TM2	TM3	TM4	TM5	TM6	TM7
TM1	-	-	-	-	-	-	-
TM2	-	-	-	-	-	-	D77-R280
TM3	-	-	-	T11-S159	-	F113-Q244	-
TM4	-	-	-	-	-	-	-
TM5	-	-	-	-	-	L198-N248	-
TM6	-	-	-	-	-	-	K251-Y278

800 ns	TM1	TM2	TM3	TM4	TM5	TM6	TM7
TM1	-	-	-	-	-	-	-
TM2	-	-	-	N72-S152	-	-	D77-R280
TM3	-	-	-	T110-S163<br>I111-S159<br>T115-I155	T121-T206	F113-Q244<br>Y114-N248	-
TM4	-	-	-	-	-	-	-
TM5	-	-	-	-	-	S188-S255	-
TM6	-	-	-	-	-	-	T243-R280<br>I232-Y288

900 ns	TM1	TM2	TM3	TM4	TM5	TM6	TM7
TM1	-	N49-L78	-	-	-	-	-
TM2	-	-	-	-	-	-	D77-R280
TM3	-	-	-	I111-S159	T121-T206	-	-
TM4	-	-	-	-	-	-	-
TM5	-	-	-	-	-	S188-S255<br>L198, G199-N248	-
TM6	-	-	-	-	-	-	-

1000 ns	TM1	TM2	TM3	TM4	TM5	TM6	TM7
TM1	-	-	-	-	-	-	-
TM2	-	-	A76-S1160	N72-S152	-	-	D77-R280
TM3	-	-	-	-	T110-T197	-	Y109-Y278
TM4	-	-	-	-	V162-T194	-	-
TM5	-	-	-	-	-	S188-R254	-
TM6	-	-	-	-	-	-	T243-R280

Table S5. Polar interactions of DRF motif in CXCR6-CXCL16 CG-MD.

0 ns		100	200	300	400	500	600	700	800	900	1000
D126	C122, V230	C122, I129	I129, V130	V130	I129, V130, C122	C122, I129, V130	*K143, I123, I129, V130	*K143, C122, V130	*K143, C122, I129, V130	*K143, C122, V130	*K143, C122, I129, V130
R127	I123, T124, V131	*L65, A142, I123, T124, T217	*I123, T124	*F69, I123	I123	I123, T124	I123	I123, V131	I123	*L65, *T66, I123	*Y138, *Y288, *S292, I123
F128	T124, V131, V132	V132, T217	T124, V131, V132	T124, V131, V132	T124, V125, V132	T123, V131, V132	-	T124, V131, V132	T124, V125, V132	T123	V125

*Interactions between sidechain of DRF residue. Residues non-polar interacts although mainchain.

Table S6. Docking scores and pIC50 of 82 antagonist of CXCR6.

Complex ID	Docking score (kcal/mol)	pIC50
F0-1	-8.02	5.237
F0-2	-7.98	4.398
F0-3	-9.46	4.585
F0-4	-9.06	4.398
F0-5	-7.83	4.398
F0-6	-8.02	4.398
F0-7	-7.65	5.237
F0-8	-8.57	5.824
F0-9	-8.32	5.022
F0-10	-8.29	4.721
F0-11	-7.52	4.398
F0-12	-8.32	4.398
F0-13	-8.53	4.398
F0-14	-7.43	4.398
F0-15	-9.32	4.398
F0-16	-7.46	6.208
F0-17	-8.24	6.854
F0-18	-8.45	6.102
F0-19	-8.08	5.678
F0-20	-8.55	6.796
F0-21	-8.23	6.194
F0-22	-7.56	6.032
F0-23	-8.68	5.796
F0-24	-8.17	6.076
F0-25	-8.08	6.387
F0-26	-8.85	6.456
F0-27	-8.44	5.745
F0-28	-7.91	5.237
F0-29	-8.82	6.796
F0-30	-8.78	6.796
F0-31	-7.48	5.947
F0-32	-8.09	4.418

F0-33	-7.8	6.456
F0-34	-8.79	5.609
F0-35	-7.39	6.215
F0-36	-7.91	4.398
F0-37	-8.98	4.301
F0-38	-8.5	4.301
F0-39	-8.14	4.301
F0-40	-8.62	4.398
F0-41	-8.5	4.398
F0-42	-7.69	4.398
F0-43	-7.27	4.398
F0-44	-9.49	4.398
F0-45	-7.88	4.398
F0-46	-8.67	4.398
F0-47	-8.32	4.398
F0-48	-8.79	4.398
F0-49	-9.58	4.398
F0-50	-8.01	4.577
F0-51	-8.17	4.076
F0-52	-8.95	4.319
F0-53	-9.79	4.31
F0-54	-8.35	4.301
F0-55	-8.75	4.292
F0-56	-9.14	4.284
F0-57	-8.28	4.301
F0-58	-7.88	4.301
F0-59	-7.7	4.301
F0-60	-7.52	5.167
F0-61	-8.11	5.022
F0-62	-7.4	4.625
F0-63	-8.79	5.469
F0-64	-8.63	5.174
F0-65	-9.83	4.625
F0-66	-9.42	4.301
F0-67	-9.89	4.301
F0-68	-10.39	6.046
F0-69	-10.26	6.523
F0-70	-10.08	5.161
F0-71	-9.94	4.979
F0-72	-9.67	4.301
F0-73	-9.37	4.896
F0-74	-9.31	6.886
F0-75	-10.74	6.77
F0-76	-8.79	6.569
F0-77	-9.88	6.585
F0-78	-9.82	6.959
F0-79	-10.71	6.602
F0-80	-10.57	5.509
F0-81	-10.08	7.097
F0-82	-8.92	7.398

Table S7. General protein-ligand nteraction model coordinates.

Name	X	Y	Z	pIC50
HydrogenAcceptor	59.3727714	65.3909095	84.6451686	0.35058078
HydrogenDonor	68.0164846	62.7024904	78.145012	0.49724503
Hydrophobic	61.415877	64.3341687	84.2736825	0.23059505
Hydrophobic	59.6718027	64.7809546	84.5175079	0.92742875
HydrogenAcceptor	64.834003	62.398006	86.2247568	0.65716015
HydrogenDonor	64.1215167	71.7991167	87.7533	0.59623049

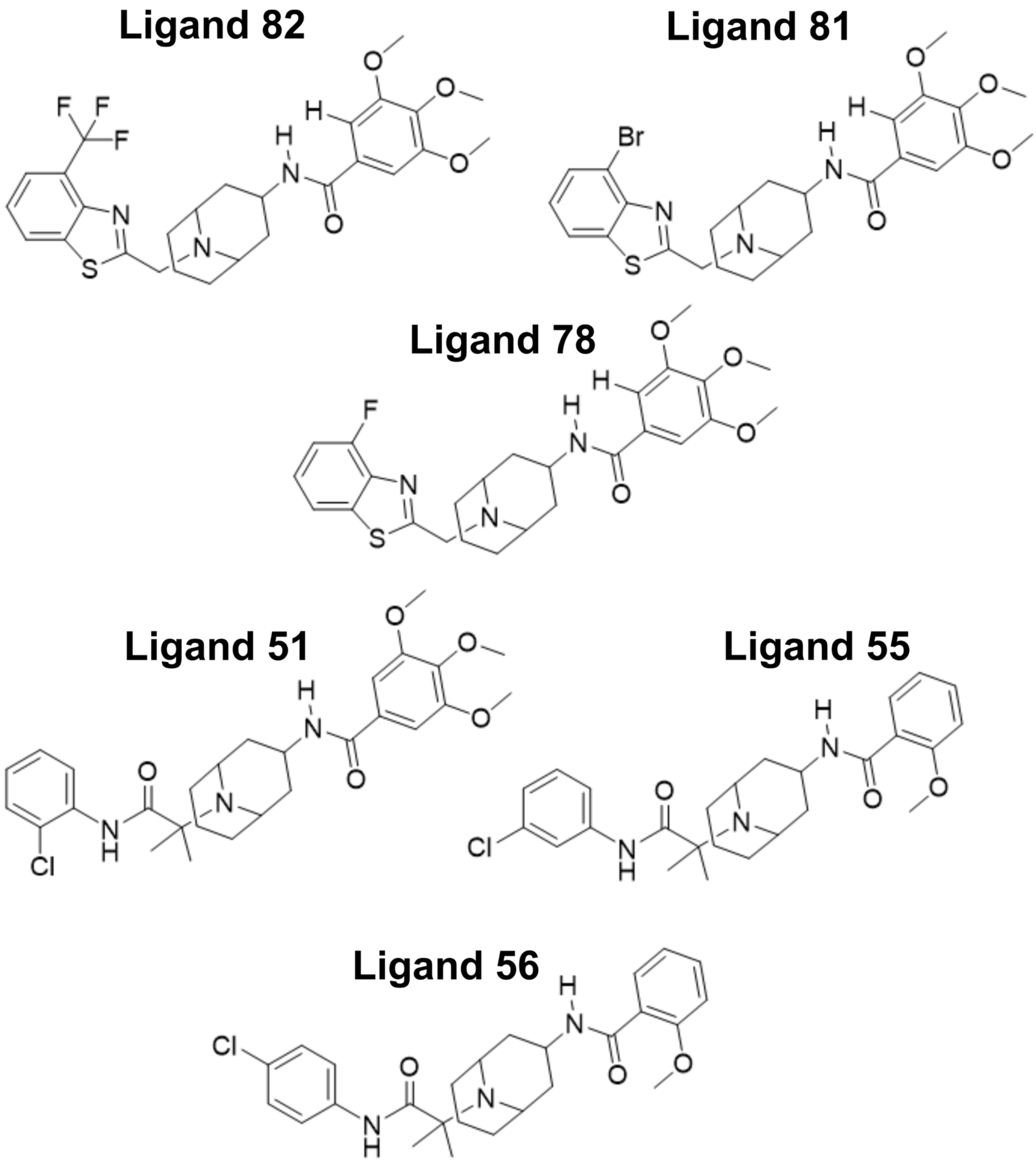

Figure S7. 2D structure of three best and three worst ligands.

Table S8. Statistical values of QSAR models.

Models	q^2	R^2	F-Test	Test-group
Model1	0.6329	0.8639	67.2740	33, 30, 39, 22, 61, 28, 36, 53, 9, 66, 56, 59, 24, 48, 72, 18,2, 52, 13, 41, 62, 46, 75
Model2	0.6066	0.8427	56.7877	52, 3, 24, 19, 29, 76, 32, 34, 28, 26, 2, 80, 59, 14, 49, 61, 53, 7, 42, 46, 81, 47, 66
Model3	0.5307	0.8076	63.7844	2, 1, 66, 52, 64, 34, 74, 65, 16, 14, 29, 32, 46, 3, 23, 62, 59, 54, 26, 59, 36, 49, 81
Model4	0.4906	0.8718	72.0608	27, 50, 25, 9, 2, 23, 14, 67, 41, 70, 33, 28, 39, 79, 66, 36, 13, 1, 71, 55, 82, 3, 76
Model5	0.5178	0.8637	67.1780	4, 80, 39, 29, 28, 37, 26, 53, 25, 58, 55, 77, 54, 23, 31, 69, 19, 7, 70, 64, 16, 81, 34
Model6	0.2146	0.8054	43.8604	18, 31, 1, 30, 77, 22, 7, 3, 71, 49, 58, 28, 79, 14, 24, 25, 55, 20, 51, 56, 17, 34, 57
Model7	0.6397	0.8731	72.8980	18, 74, 38, 2, 72, 80, 28, 52, 16, 15, 12, 73, 70, 26, 47, 13, 32, 54, 65, 62, 59, 79, 56
Model8	0.5430	0.8624	66.4102	6, 69, 80, 44, 36, 55, 58, 39, 15, 7, 46, 24, 41, 53, 32, 51, 29, 45, 82, 78, 20, 79, 40
Model9	0.5340	0.8479	59.1133	20, 79, 42, 29, 40, 75, 9, 77, 41, 28, 19, 59, 15, 62, 50, 43, 69, 17, 31, 53, 21, 76, 66
Model10	0.5404	0.8264	50.4763	23, 52, 30, 31, 14, 24, 32, 22, 16, 27, 3, 65, 72, 36, 46, 45, 51, 15, 6, 55, 12, 43, 75
